# Mitochondrial Function Are Disturbed in the Presence of the Anticancer Drug, 3-Bromopyruvate

**DOI:** 10.3390/ijms22126640

**Published:** 2021-06-21

**Authors:** Magdalena Cal, Irwin Matyjaszczyk, Karolina Filik, Rafał Ogórek, Young Ko, Stanisław Ułaszewski

**Affiliations:** 1 Department of Mycology and Genetics, University of Wroclaw, 51-148 Wroclaw, Poland; irwin.matyjaszczyk@uwr.edu.pl (I.M.); rafal.ogorek@uwr.edu.pl (R.O.); stanislaw.ulaszewski@gmail.com (S.U.); 2 Laboratory of Medical Microbiology, Department of Immunology of Infectious Diseases, Hirszfeld Institute of Immunology and Experimental Therapy, Polish Academy of Sciences, 53-114 Wroclaw, Poland; karolina.filik@hirszfeld.pl; 3 KoDiscovery, LLC, Baltimore, MD 21202, USA; youngheeko@kodiscovery.org

**Keywords:** 3-bromopyruvate, mitochondria, superoxide generation, oxidative stress, mtDNA damage, yeast

## Abstract

3-bromopuryvate (3-BP) is a compound with unique antitumor activity. It has a selective action against tumor cells that exhibit the Warburg effect. It has been proven that the action of 3-BP is pleiotropic: it acts on proteins, glycolytic enzymes, reduces the amount of ATP, induces the formation of ROS (reactive oxygen species), and induces nuclear DNA damage. Mitochondria are important organelles for the proper functioning of the cell. The production of cellular energy (ATP), the proper functioning of the respiratory chain, or participation in the production of amino acids are one of the many functions of mitochondria. Here, for the first time, we show on the yeast model that 3-BP acts in the eukaryotic cell also by influence on mitochondria and that agents inhibiting mitochondrial function can potentially be used in cancer therapy with 3-BP. We show that cells with functional mitochondria are more resistant to 3-BP than *rho*^0^ cells. Using an MTT assay (a colorimetric assay for assessing cell metabolic activity), we demonstrated that 3-BP decreased mitochondrial activity in yeast in a dose-dependent manner. 3-BP induces mitochondrial-dependent ROS generation which results in ∆*sod2*, ∆*por1*, or ∆*gpx1* mutant sensitivity to 3-BP. Probably due to ROS mtDNA lesions rise during 3-BP treatment. Our findings may have a significant impact on the therapy with 3-BP.

## 1. Introduction

3-bromopyruvate (3-BP) is an analog of pyruvic acid. Many studies published on 3-BP indicate that after entering the cell (by monocarboxylate transporters—MCTs in human cancer cells [1,2] and through the lactate/pyruvate H^+^ symporter Jen1 in yeast [3]) it turns out to have a pleiotropic effect. This effect results from numerous activities, where the best from described is the effect on metabolism by inhibition of key glycolytic enzymes including hexokinase II [4,5,6,7], glyceraldehyde 3-phosphate dehydrogenase (GAPDH) [8], and lactate dehydrogenase (LDH). It has also been proved that some mitochondrial functions are disturbed in the presence of 3-BP, e.g., by inhibiting complexes I and II [9]. Interestingly, in the presence of 3-BP, the level of ATP drops sharply [9]. The presence of 3-BP also leads to an increase in reactive oxygen species (ROS) [10,11,12,13] which, together with a strong reduction of glutathione, leads to oxidative stress [14,15,16,17]. Because of numerous activities, especially strong inhibition of glycolytic enzymes, 3-BP is a promising anti-cancer compound. Many cancer cells exhibit the Warburg effect, namely enhanced glycolysis even in the presence of oxygen. As a consequence, the Warburg effect leads to overproduced MCTs to efflux the excess of lactic acid [18]. Described mechanisms explain why the concentrations of 3-BP that are not toxic for normal cells are strongly toxic to tumor cells, making 3-BP a promising candidate for anti-cancer therapy [19]. Previous research conducted on animal models [20], multiple myeloma cancer cell lines (MM cells) [21], yeast *Saccharomyces cerevisiae*, and yeast-like fungi *Cryptococcus neoformans* cells [16] showed that in the presence of 3-BP, an increase in reactive oxygen species (ROS) occurs in the all above-mentioned cell types [16,22,23,24]. The presence of increased amounts of ROS as a result of 3-BP activity in the cell may be a possible cause of DNA strand damage [24]. Probably, observed glutathione level decrease upon 3-BP treatment on fungal and algal cells is the result of glutathione-3-BP complex formation [15,16,17]. In addition, depletion of glutathione pools can lead to an increase in ROS levels.

Mitochondria are two membranes organelles found in most eukaryotic cells which have multiple functions important to proper cell activity. One of the most important functions of mitochondria is the supply of cellular energy or participation in the production of amino acids. Recent studies suggest that the essential function of mitochondria is to allow aspartate synthesis, as it was shown that supplementation of aspartate restores the growth of respiratory deficient mammalian cells [25]. The other functions are for example regulation of apoptosis, generation of reactive oxygen species (ROS) [26], or iron-sulfur cluster biogenesis [27]. In many studies, mitochondria are described as the “energy factories” because of ATP synthesis through the tricarboxylic acid cycle (TCA) and oxidative phosphorylation (OXPHOS) [27,28]. Yeast are facultative anaerobes which means that when grown on a medium with a non-fermentable carbon source (e.g., ethanol or glycerol) the mitochondrial respiratory function is indispensable for yeast to survive. TCA cycle is carried out in the matrix of mitochondria and is the second stage of cellular respiration. 3-BP disrupts the proper functioning of the enzymes in this pathway (isocitrate dehydrogenase, α-ketoglutarate dehydrogenase, and dehydrogenase succinate) [29,30] which in turn leads to disturbances in the functioning of the citric acid cycle and limitations in the production of energy. The respiratory chain responsible for oxidative phosphorylation consists of four complexes (I, II, III, and IV) which transports electrons to a vectorial proton transport across the mitochondrial membrane, and the complex V, (F1Fo-ATP synthase), which can use the generated electroosmotic potential to synthesize ATP from ADP [31]. Additionally, the reduction of O_2_ to H_2_O is continuously carried [32]. When electrons leak from the respiratory chain the reduction of oxygen is incomplete which leads to the formation of superoxide anions (O_2_^−^) that are metabolized by SOD enzymes to hydrogen peroxide (H_2_O_2_) which can produce hydroxyl radicals (OH•) in Fenton’s reaction. ROS may play a role in physiological cellular processes such as apoptosis or can be signaling molecules in redox biology [27,33]. Reactive oxygen species can damage DNA, proteins, or other biological molecules. The level of free radicals in the cell are maintained by antioxidant enzymes, like superoxide dismutases Sod1 and Sod2 or glutathione (Gsh) [34,35]. An excess of free radicals leads to oxidative stress and may, as a consequence, lead to an adverse effect on the cell. Disturbances in the proper functioning of mitochondria are associated with extremely severe consequences for the cell: e.g., lack of cellular energy, excess free radicals, or disturbances in the repair of damaged DNA. We previously showed [24] that a sufficiently low concentration of 3-BP can induce cytotoxicity at the nuclear genome level in yeast. The main goal of our research was to investigate, whether 3-BP is also a genotoxic agent for *S. cerevisiae* mitochondrial DNA (mtDNA) under the conditions where most cells are still viable. We showed in this work that 3-BP leads to generated mitochondrial-mediated ROS, mtDNA lesions, and that 3-BP impaired mitochondrial function. Significantly, a better understanding of the mechanism of 3-BP’s action and its influence on the cell can lead to improved treatment methods.

## 2. Results

### 2.1. The Role of Functional Yeast Mitochondria in Response to 3-BP—Viability and Sensitivity of W303-1A and rho^0^ Strains to 3-BP

**Figure 1:**
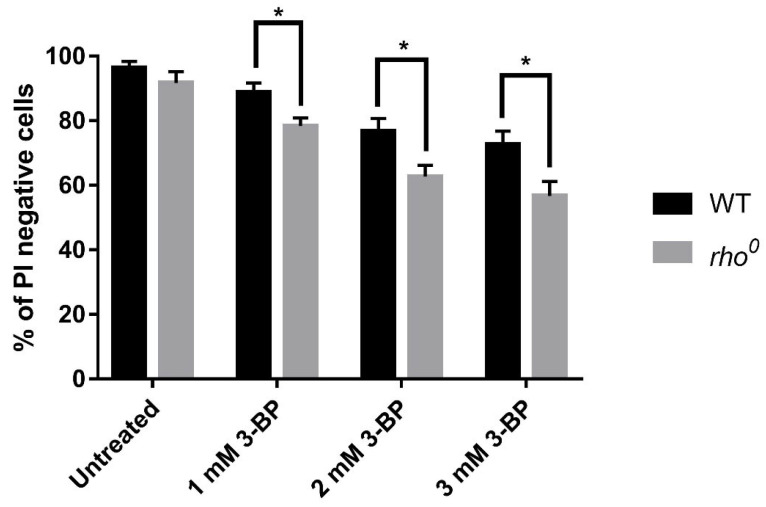
3-BP causes cell death in yeast W303-1A (WT) and *rho*^0^ strain. Logarithmically growing W303-1A (WT) and *rho*^0^ cells were treated for 4 h with different 3-BP concentrations, incubated with PI, and viewed under a fluorescence microscope. Percent of PI-positive cells was calculated. * *p* ≤ 0.05.

**Figure 2:**
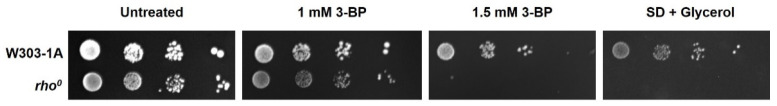
*rho*^0^ mutants exhibit increased sensitivity to 3-BP. Spot tests were performed to test the sensitivity of *rho*^0^ and WT strain to 3-BP. Serial dilutions of strains were spotted on SD medium plates with different 3-BP concentrations and incubated for 2 days at 30 °C.

Experiments were performed in SD minimal medium with sucrose as a carbon source to allow *JEN1* expression which is responsible for 3-BP uptake [3]. Due to the glucose repression mechanism, *JEN1* is not expressed in the presence of glucose in the medium. To check the sensitivity of W303-1A and *rho*^0^ strains to 3-BP we performed Propidium Iodide (PI) staining (Figure 1). Necrotic cells with damaged membranes are stained by propidium iodide while healthy cells remain impenetrable to the dye [36]. Yeast cells (W303-1A and *rho*^0^) were treated with various concentrations of 3-BP (1 mM, 2 mM, and 3 mM) and stained with propidium iodide (PI) (Figure 1). PI staining revealed that incubation with 3-BP for 4 h induced necrosis in both strains in a dose-dependent manner, *rho*^0^ strain was more sensitive to 3-BP than the W303-1A strain (Figure 1). Determination of viability is also necessary for further experiments to choose concentrations of 3-BP at which most of the cells remain viable. We performed also a spot test to check the sensitivity of both strains to 3-BP to confirm results from PI staining. In agreement with earlier results, the *rho*^0^ strain is more sensitive to 3-BP (Figure 2). Taken together these results indicated that functional mitochondria are important for survival in presence of 3-BP.

### 2.2. 3-BP Disturbs Mitochondrial Function and Reduces Metabolic Activity in Yeast

#### *S. cerevisiae* Cells

**Figure 3:**
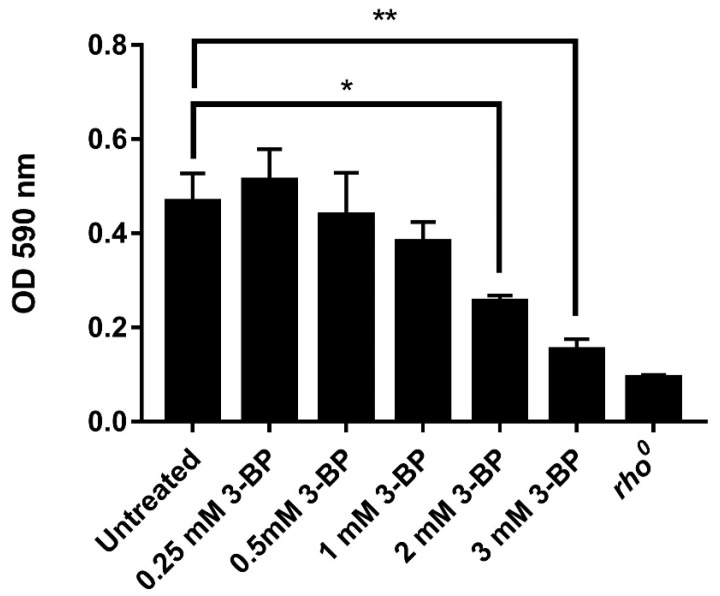
3-BP leads to inhibition of mitochondrial activity in yeast. Logarithmically growing W303-1A (WT) cells were treated for 2 h with different 3-BP concentrations and collected. OD600 was adjusted to 0.5 in PBS and cells were incubated for 4 h in presence of 0.5 mg/mL MTT. * *p* ≤ 0.05; ** *p* ≤ 0.01.

To test how 3-BP treatment affects *rho*^0^ cells, we checked the metabolic activity of cells using this MTT assay. This assay was designed to measure the conversion of the tetrazolium salt to water-insoluble formazan in a living cell. The rate of conversion depends on the metabolic activity of the cell as MTT is metabolized to formazan by dehydrogenases in the mitochondria and it is worth noting that enzymes outside mitochondria could also contribute to formazan formation [37]. W303-1A and *rho*^0^ cells were incubated in SD medium with various 3-BP concentrations for 2 h, incubated for 4 h with MTT reagent and formazan was solubilized with DMSO absorbance values of samples were measured at 590 nm (Figure 3). We observed a strong reduction in absorbance with increasing 3-BP concentrations (0.25 mM, 0.5 mM, 1 mM, 2 mM, 3 mM), which suggests that 3-BP strongly reduces formazan formation and metabolic activity of cells. 3 mM concentration showed the greatest reduction in signal nearing reading of *rho*^0^ strain (Figure 3).

### 2.3. Exposure to 3-BP Leads to Generation of Superoxide Anion in Mitochondria

**Figure 4:**
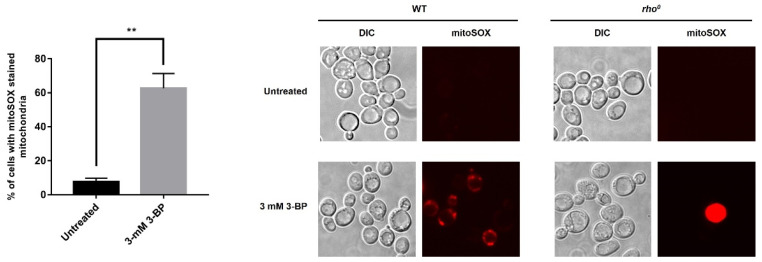
3-BP induces generation of superoxide in mitochondria. Mitochondrial superoxide level was measured with a MitoSOX fluorescent probe. WT cells were treated with different 3-BP concentrations for 4 h and loaded with MitoSOX Red, signal was detected under a fluorescence microscope. The percentage of cells with a mitochondrial signal was calculated. Yeast cells that exhibit fluorescence from the whole-cell represent dead cells and were not taken into account. ** *p* ≤ 0.01.

To find what causes the toxic activity of 3-BP towards mitochondria, it was decided to check whether there is the production of mitochondria-dependent free radicals in the presence of 3-BP. It was shown that leakage of electrons from the mitochondrial respiratory chain is the main reason for the accumulation of endogenous ROS in the yeast *S. cerevisiae* [38]. We also know that *S. cerevisiae* can function without mtDNA [38], so we can further investigate the role of the respiratory chain in dealing with stress induced by the presence of 3-BP in the cell. We used MitoSOX™ Red because it is a fluorescent dye specifically targeted to mitochondria in live cells which is oxidized by superoxide producing a fluorescent product that binds to nucleic acids, to find out if 3-BP induced generation mitochondria-dependent superoxide (Figure 4). W303-1A and *rho*^0^ cells were incubated with 3 mM 3-BP for 4 h, loaded with MitoSOX Red, and the signal was observed under a fluorescent microscope (Figure 4). This detection method showed the greatest sensitivity as the signal from mitochondria is very faint. In the untreated W303-1A strain, a very weak signal was detected in up to 10% of cells. In cells incubated with the 3-BP majority of cells (63%) showed strong signals from mitochondria (Figure 4). In the negative control *rho*^0^ strain, no mitochondrial fluorescence was detected in untreated and 3-BP treated cells (Figure 4). This finding suggests that 3-BP induces the generation of superoxide anion and that mitochondria are the source of 3-BP induced ROS production.

### 2.4. 3-BP Induces Lesion Formation in mtDNA in Yeast

**Figure 5:**
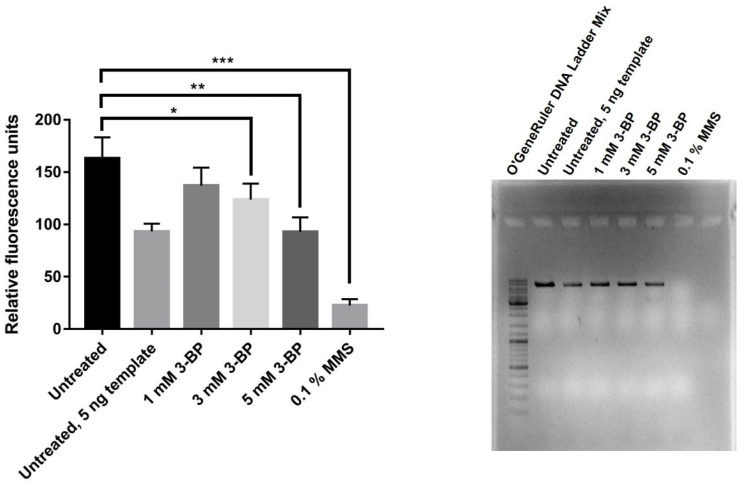
3-BP causes an increase in mtDNA lesions detected in the QPCR assay. Logarithmically growing WT cells were treated with indicated 3-BP concentrations and 0.1% MMS for 5 h and DNA was extracted from cells. DNA concentrations were adjusted and PCR was run to amplify 6.9 kb fragment of mitochondrial *COX1* gene. dsDNA content was measured with QuantiFluor (Promega). PCR products were visualized with agarose gel electrophoresis. * *p* ≤ 0.05; ** *p* ≤ 0.01; *** *p* ≤ 0.001.

Taking into account the fact that 3-BP induces a high level of mitochondrial-dependent oxygen radicals (Figure 4) and that we observed nuclear DNA damage upon 3-BP treatment [24], we decided to check whether mtDNA is damaged. QPCR amplifies a large fragment (6.9 kb) of the mitochondrial *COX1* gene. DNA damage such as strand breaks, adducts, etc. inhibits polymerase progression in PCR reaction and lowers the yield of the reaction. Reduction in amplification can be quantified with dsDNA-specific fluorescent dye such as QuantiFluor^®^ dsDNA Dye (Promega, Madison, WI, USA). Reduction in the quantity of PCR product suggests that there was a reduction in the total number of mtDNA in the sample or that mtDNA was damaged. W303-1A strain was incubated with different 3-BP concentrations and 0.1% MMS as a positive control for 5 h, total DNA was extracted and QPCR was performed (Figure 5). Quantification of product revealed that 3-BP caused a reduction in PCR yield in the dose-dependent mechanism. Reaction with half the amount of template (5 ng) used as a control for reaction gave half the amount of PCR product. In the sample treated with DNA methylating agent, the MMS reaction was inhibited completely. Agarose gel electrophoresis confirmed that the correct size product was obtained (Figure 5). Results indicate that 3-BP reduces mtDNA copy number and/or induces the formation of lesions in mtDNA.

### 2.5. Compounds Inhibiting Electron Transport Chain (ETC) and Inducing Oxidative Stress Work Synergistically with 3-BP

**Figure 6:**
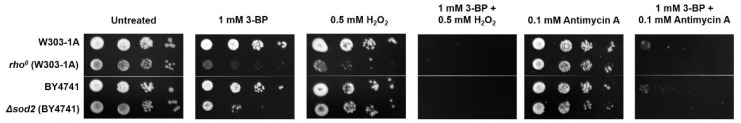
3-BP works synergistically with agents inducing oxidative stress and inhibiting mitochondrial ETC (electron transport chain). Spot tests were performed to test the sensitivity of wild type (BY4743, W303-1A), *rho*^0^, Δ*sod*2 to 3-BP in combination with Antimycin-A and H_2_O_2_. Serial dilutions of strains were spotted on SD medium plates with different concentrations of compounds and incubated for 2 days at 30 °C.

To test how inhibition of ETC and oxidative stress can affect susceptibility to 3-BP we performed spot tests where W303-1A, *rho*^0^, and Δ*sod2* strains were grown with 3-BP in combination with antimycin A and H_2_O*_2_* (ETC inhibitor at complex III and oxidative stress-inducing agent, respectively). Both agents increased the sensitivity of tested strains to 3-BP (Figure 6).

### 2.6. Role of mtDNA Maintenance, mtDNA Repair Proteins and Antioxidant Enzymes in Tolerance to 3-BP

**Figure 7:**
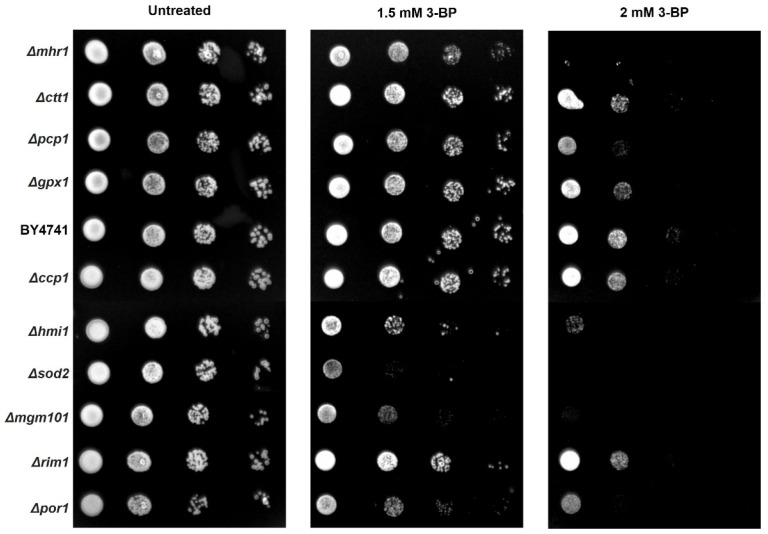
Deletion mutants strains of mtDNA maintenance, repair, and ROS protection genes exhibit increased sensitivity to 3-BP. Serial dilutions of strains were spotted on SD medium plates with different 3-BP concentrations and incubated for 2 days at 30 °C.

We tested how deletion of genes encoding mtDNA maintenance, mtDNA repair proteins, and antioxidant enzymes affects the sensitivity of strains to 3-PB in spot test assay (Figure 7). Δ*mgm101* [39], Δ*hmi1* mutants which lack genes responsible for mtDNA maintenance display increased sensitivity to 3-BP compared to W303-1A strain, which is in agreement with increased sensitivity of *rho*^0^ strain and delineates the importance of mtDNA for survival in presence of 3-BP (Figure 7). In addition, deletion of *mhr1* gene encoding protein involved in the regulation of mitochondrial DNA recombination and repair [40] reduces tolerance to 3-BP. In contrast deletion of *rim1* gene encoding protein involved in mtDNA replication [41] did not affect the viability of strain in presence of 3-BP. Deletion of antioxidant enzyme genes: mitochondrial superoxide dismutase (*SOD2*), cytosolic catalase (*CTT1*), and glutathione peroxidase (*GPX1*) increase sensitivity to 3-BP, which suggest an important role of antioxidant enzymes in tolerance to 3-BP. Deletion mutant of mitochondrial cytochrome-c peroxidase (∆*ctt1*) did not show significantly reduced viability compared to W303-1A strain (Figure 7).

## 3. Discussion

**Figure 8:**
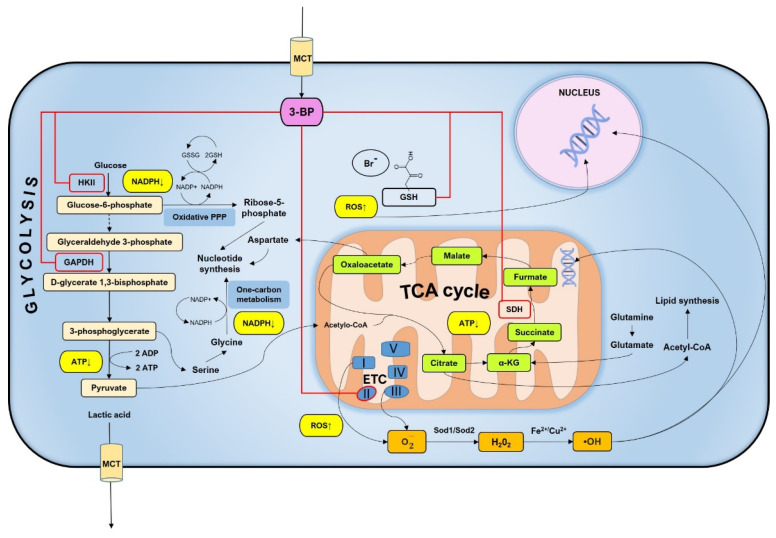
Possible mechanisms of 3-BP’s killing actions to cells. After entering the cell through MCTs’ channels, 3-BP inhibits glycolytic enzymes (i.e., HK2, GAPDH) and mitochondrial oxidative phosphorylation pathway (i.e., complex II/SDH). Additionally, 3-BP leads to a reduction in the amount of glutathione while increasing the level of ROS. By disrupting the mitochondrial electron transport chain, 3-BP leads to the generation of mitochondrial-dependent ROS, which results in mtDNA damage. Excess ROS may potentially induce damage to genomic DNA.

Full understanding of the mechanisms of potential drugs is extremely important in the conduct of therapy. 3-BP is a compound with the potential to be an effective anti-cancer drug that has already been characterized in many aspects (Figure 8) [1,2,42,43]. It is known from previous studies that 3-BP affected several glycolytic and mitochondrial enzymes in the cell and shows strong activity against many cancers [4,7,8,9,42]. Importantly, 3-BP is a compound that is toxic primarily to cancer cells, which impacts their metabolism and the large number of MTCs—mono-carboxylate transporters in the membrane by which 3-BP enters the cell [1,2]. It has also been proved that 3-BP leads to rapid depletion of ATP level [3,9]. In our previous research work, we have shown that 3-BP is a genotoxic agent and leads to induction of DNA damage both in yeast and human cancer cells, potentially through reactive oxygen species [24]. The production of cellular energy in the form of ATP, the proper functioning of the respiratory chain, or participation in the production of amino acids is one of many important functions of mitochondria [26,27,28,44]. It has been shown that mitochondria are essential in the cellular response to many stress factors, such as oxidative stress or the presence of compounds such as arsenic or lead [45,46]. Mitochondrial dysfunction can cause cellular imbalance and thus lead to disease [38]. Here we continue our research on the genotoxicity of 3-BP and show for the first time in *S. cerevisiae* cells that 3-BP induces free radical formation that is dependent on mitochondria. We also observe mtDNA damage and mitochondrial dysfunction upon 3-BP treatment. We show that mitochondria are the target of 3-BP action and, on the other hand, that the organelles are important for the cellular response to 3-BP action.

First of all, we checked the viability of wild-type (W303-1A) and mitochondrial DNA (*rho*^0^) deficient *S. cerevisiae* strains in the presence of 3-BP (Figure 1). After 4 h treatment, we could observe decreased viability of *rho*^0^ strain in all tested compound concentrations which were seeded with increasing concentration of 3-BP (Figure 1). It was assumed that the presence of functional mitochondria in baker’s yeast cells was essential for their survival in the presence of 3-BP. Additionally, it was shown that the *rho*^0^ strain is very sensitive even at a 3-BP concentration of 1.5 mM (Figure 2), which is in line with the previous conclusion. In a similar concentration, 2 mM of 3-BP, the survival of *rho*^0^ is at the level of 65–70% (Figure 1), while the mitochondrial activity and the overall metabolic activity of the cell decrease rapidly (Figure 3) which may consequently lead to growth and division disorders and the observed strong sensitivity. It seems that cells with functional mitochondria are more resistant to 3-BP than *rho*^0^ cells. Due to the results described above, we decided to check if and at what level 3-BP leads to disturbances in the activity of mitochondria (Figure 3). For this purpose, we treated the wild type W303-1A baker’s yeast strain at various concentrations of 3-BP and observed a decrease in mitochondrial activity, almost to the level of the *rho*^0^ mutant, with increasing 3-BP concentration (Figure 3). At a concentration of 2 mM 3-BP, where the survival rate of the strain was 78% (Figure 1), the decrease in mitochondrial activity was already significant (Figure 3).

To determine what causes the toxic activity of 3-BP towards mitochondria, it was decided to check whether there is damage to mtDNA and the production of mitochondria-dependent free radicals in the presence of 3-BP. As shown in Figure 4, there is a significant increase in the percentage of cells with the signals from mitochondria after 3-BP treatment, about 6 times higher level of superoxide upon 3-BP treatment than observed in wild type without 3-BP (Figure 4). Literature data show that the superoxide anion is produced in the first place and in the greatest amount when the respiratory chain is non-functional and electrons leak from the electron transport chain. Next, the superoxide is converted to H_2_O_2_ by superoxide dismutase Sod2 [47]. We have also previously shown that 3-BP induces oxidative stress which may be the cause of DNA damage [24], here we demonstrate that an important source of these 3-BP induced free radicals in mitochondria. This is further supported by the sensitivity of the Sod2 deletion mutant (Figure 6) to 3-BP. These results suggest that probably mitochondria are one of the important ROS sources in 3-BP treated cells, likely due to impairment of ETC. Deletions mutant strains of genes involved in detoxification of ROS (Sod2, Ctt1, Gpx1) exhibit increased sensitivity to 3-BP (Figure 7) which confirms that this compound can lead to induction of ROS production. Even sensitivity to 3-BP of the ∆*por1* mutants (Figure 7, Por1-mitochondrial porin, couples the glutathione pools) may indicate that 3-BP induces the formation of mitochondrial-dependent free radicals, mainly superoxide radicals, which in the absence of important antioxidant enzymes, may disrupt the functioning of the mitochondria and the entire cell. Oxidative stress induction has also been demonstrated in cancer cell lines [11]. This could also be explained by the mechanism of action of 3-BP, as it was shown to cause depletion of major ROS scavenger, glutathione [17]. The results presented in this work are in agreement both with the quoted literature data [38,47] and are an extension and complement to our previous work [24]. We demonstrated that 3-BP leads to oxidative stress and DNA damage in yeast cells. It is known that oxidative stress can cause DNA damage. Taking into account the fact that 3-BP induces a high level of mitochondrial-dependent oxygen radicals (Figure 4) and that we observed nuclear DNA damage with 3-BP treatment [24], we decided to check whether mtDNA is damaged. In *S. cerevisiae* mitochondria dysfunction can lead to nuclear mutator phenotype [48]. This is in accordance with our earlier results which indicate that 3-BP caused DNA damage [24]. Reactive oxygen species can cause various types of mtDNA damage, such as single-strand breaks, double-strand breaks, or oxidative damage to nitrogen bases [49,50,51]. Mitochondrial DNA is more prone to mutation than nuclear DNA. Mutations in mtDNA have an impact on the proper functioning of the mitochondria and the entire cell, leading to disturbances in many processes such as the respiratory chain and the generation of cellular energy. Mutations in mtDNA are associated with many diseases such as neurological disorders, age-related diseases, and several cancers [44]. As we suspected and in accordance with the literature data it was shown that with 3-BP treatment there is an increase in mtDNA lesions detected in qPCR (Figure 5). As the concentration of 3-BP increased, an increase in mtDNA instability was noted (Figure 5). This result confirmed the hypothesis that mitochondria might be, outside the glycolytic pathway, direct targets of 3-BP—induced toxicity in eukaryotic cells. On the other hand, 3-BP by influence on energy metabolism and mitochondrial function may cause the reduction of mtDNA content by reducing the number of mitochondria by mitophagy. We have also shown that 3-BP works synergistically with other compounds which are known to induce oxidative stress and inhibit the respiratory chain such as H_2_O_2_ and antimycin A (Figure 6). Probably the observed synergistic sensitivity results from the fact that 3-BP leads to damage of mitochondrial function, disturbance of ATP level, which in consequence lead to increased sensitivity of cells to oxidative stress which is induced by H_2_O_2_ and antimycin A (Figure 6). It is very interesting to note that both mutant lacking mtDNA (*rho*^0^) and mutant lacking important antioxidative enzymes—superoxide dismutase (∆*sod2*) show high sensitivity to 3-BP (Figure 6), which proves that free radicals in the presence of 3-BP are induced in mitochondria and in a situation where there is no antioxidant enzyme to transform superoxide to hydrogen peroxide, the strains are as sensitive as in the absence of mtDNA (Figure 6). These results are in agreement with the sensitivity of deletion mutant test of strains lacking antioxidant enzymes and *rho*^0^ strain (Figure 6) displaying dysregulated functional electron transport chain (ETC) and membrane potential. In addition, and on the other hand, antimycin A, an inhibitor of ETC, may increase the sensitivity of cells to 3-BP supporting the idea that functional mitochondria are crucial for survival in presence of 3-BP. It would be interesting to use 3-BP in anti-cancer therapy together with other compounds which induce oxidative stress and inhibit the respiratory chain. Increased sensitivity of *rho*^0^ strain can be explained by the fact that (1) mitochondrial function is crucial for resistance against oxidative stress [52], (2) *rho*^0^ have reduced mitochondrial membrane potential (MMP) leading to impairment of iron-sulfur synthesis [53], (3) inhibition of TCA cycle leads to interruption of dNTPs and amino acid synthesis (Figure 8). We also analyzed the effect of 3-BP on null mutants deleted in nuclear genes involved in a variety of mitochondrial functions, especially those that are involved in the repair and maintenance of mtDNA damage (Mhr1, Pcp1, Hmi1, Mgm101) and ROS protection (Sod2, Por1) (Figure 7). This analysis also confirmed that proteins important for mtDNA stability and mtDNA repair are needed in the presence of 3-BP because probably the presence of 3-BP in the cell induces a cellular response to mtDNA damage and may lead to mtDNA stability disturbance (Figure 7). Both, gene *MGM101* deficient strain (∆*mgm101*) and ∆*mhr1* deletion mutant show high sensitivity to 3-BP (Figure 7). Both Mgm101 and Mhr1 are involved in the recombinational repair of mtDNA damage and in maintaining the stability of mtDNA. Mhr1 prevents mtDNA instability [40]. Mgm101 is also a well-characterized protein that is involved in the repair and replication of the mitochondrial genome [54]. These results indicate that lack of these genes results in increased sensitivity to 3-PB which could imply that maintenance and repair of mtDNA are important in contest of cellular response to 3-BP. These proteins are critical for the viability of the cells in the presence of 3-BPActivity of 3-BP may also disturb the stability of the mtDNA and consequently the mtDNA replication processes, whereby these proteins are essential. The sensitivity of subsequent deletion mutants, ∆*pcp1* and ∆*hmi1*, confirms that mitochondrial DNA instability occurs in the presence of 3-BP, as both proteins are involved in the maintenance of mitochondrial DNA. Pcp1 protein is a serine protease that processes L-Mgm1 into s-Mgm1. These two isoforms are essential for normal mitochondrial morphology and the maintenance of mDNA stability [55]. Similarly, Hmi1 is a helicase required for the maintenance of the mitochondrial genome [56]. Disruption of mitochondrial ETC causes the generation of ROS [57] and subsequent oxidative damage which can lead to destabilization of the mitochondrial and nuclear genome [38]. Probably all the effects we observe result from the pleiotropic action of 3-BP in the cell (Figure 8), however, these results confirm all previously described and enhance the probability of induction of mtDNA instability upon 3-BP treatment, probably because of oxidative stress (Figure 4) and damage of mtDNA (Figure 5).

Here we show the new action of 3-BP as a genotoxic agent for *S. cerevisiae* mitochondrial DNA (mtDNA). 3-BP leads to generated mitochondrial-mediated ROS and impaired mitochondrial function. What we show is that cellular interactions involved in the 3-BP response included a response to DNA damage and oxidative stress. In the presence of 3-BP, yeast cells become extremely sensitive to oxidative stress. 3-BP treatment leads to oxidative stress, when we introduce another source of ROS to a cell with already compromised antioxidative potential, it could lead to a lethal effect. This may have implications in the design of 3-BP therapy in combination with oxidative stress-inducing agents. Results presented in this work are in agreement with previous findings which indicate that cancer cells grown under hypoxic conditions are more sensitive to 3-BP than those grown under normoxic conditions. Furthermore, it was demonstrated that respiratory deficient cancer cell lines were more susceptible to 3-BP [58]. Due to the anti-tumor activity of 3-BP, it is extremely important to know the full activity of the potential anti-tumor drug 3-BP in the cell. This knowledge may contribute to a better understanding of the effects that 3-BP can have during therapy and may lead to therapy improvement.

## 4. Materials and Methods

### 4.1. The Yeast Strains, Growth Conditions and Cell Treatment

*Saccharomyces cerevisiae* strains were grown in SD minimal medium [0.67% Yeast Nitrogen Base (Becton, Dickinson & Company^®^, Franklin Lakes, NJ, USA), 2% sucrose (Chempur^®^, Piekary Śląskie, Poland)] supplemented with amino acids [5 mg/mL adenine, 2 mg/mL histidine, 6 mg/mL leucine, 4 mg/mL tryptophan, 2 mg/mL uracil (Sigma-Aldrich^®^, St. Louis, MO, USA)], with addition of 1.5% agar (Becton, Dickinson & Company^®^) for solid medium. For treatment with compounds, cells were grown to mid-logarithmic phase (OD600 0.4–0.6) at 30 °C. The yeast strains used in this study are listed in Table 1.

To prepare *rho*^0^ strain W303-1A yeast were grown at 30 °C at 200 RPM in YPD medium o mid-logarithmic phase (OD600 0.5–0.8), ethidium bromide was added to the medium to 30 μg/mL final concentration, and cells were incubated for 24 h at 30 °C 200 rpm. Following incubation, cells were plated on a YPD medium to obtain single colonies and incubated for 3 days at 30 °C. Obtained colonies were plated on a YPGly medium containing glycerol as a carbon source to identify respiratory deficient cells. To verify loss of mtDNA, strains displaying no growth on YPGly plates were grow cells to mid-log phase, DAPI (Sigma-Aldrich, St. Louis, MO, USA) was added to a final concentration of 5 μg/mL, cells were incubated at 30 °C 200 rpm for 30 min, washed with PBS, and observe under a fluorescent microscope.

### 4.2. Cell Viability Assay—Propidium Iodide (PI) Staining

Propidium Iodide (PI) is a fluorescent dye that can pass through damaged membranes of dead cells and intercalate within the DNA staining dead cells which can be detected with a fluorescence microscope or flow cytometer. Logarithmic phase cell culture of W303-1A and *rho*^0^ strains in SD medium were treated with 3-BP. After treatment OD600 of samples was adjusted to 1.0 and 1 mL of cells was centrifuged (1 min, 10,000× *g*), the medium was removed, and the cell pellet was resuspended in 1 mL of PBS. Propidium Iodide (Sigma-Aldrich, St. Louis MO, USA) was added to a final concentration of 2 μg/mL and samples were incubated for 15 min in the dark. Samples were centrifuged (1 min, 10,000× *g*), the supernatant was removed and cells were resuspended in 1 mL of PBS (Phosphate Buffered Saline). Fluorescence was detected with Axio Imager M1 epifluorescence microscope (Carl Zeiss, Jena, Germany) using a CFP filter set. Percent of PI-positive cells was calculated.

### 4.3. Yeast Sensitivity Test (Spot Test)

To test the sensitivity of deletion mutants to compounds spot tests were performed. Individual strains were transferred from fresh SD agar plates to SD medium and OD600 of cell suspension was adjusted to 0.25, 10-fold serial dilutions were made (10^−1^, 10^−2^, and 10^−3^) and 5 µL was spotted onto SD agar containing tested compounds. Plates were incubated for 3–5 days at 30 °C.

### 4.4. Mitochondrial ROS Measurements

MitoSOX™ Red is a fluorescent dye specifically targeted to mitochondria in live cells which is oxidized by superoxide producing a fluorescent product that binds to nucleic acids. Logarithmic phase cell culture of W303-1A and *rho*^0^ strains in SD medium were treated with 3-BP. After treatment OD600 of samples was adjusted to 1.0 and 1 mL of cells was centrifuged (1 min, 10,000× *g*), the medium was removed, and the cell pellet was resuspended in 1 mL of PBS. Freshly prepared MitoSOX Red (Invitrogen™, Waltham, MA, USA) was added to a final concentration of 5 μM and samples were incubated for 15 min in the dark. Samples were centrifuged (1 min, 10,000× *g*), the supernatant was removed and cells were resuspended in 1 mL of PBS. Fluorescence was detected with Axio Imager M1 epifluorescence microscope (Carl Zeiss, Jena, Germany) using a CFP filter set. Percent of cells exhibiting signal from mitochondria was calculated, *rho*^0^ strain was used as a negative control.

### 4.5. Quantitative Polymerase Chain Reaction

QPCR amplifies 6.9-kb mitochondria fragments in the *COX1* gene. Damaged DNA such as strand breaks, adducts, etc. can lead to inhibition of polymerase in PCR reaction which results in a lower yield of the reaction. To increase the sensitivity of the assay large gene fragments are amplified. Reduction in amplification can be quantified with a dsDNA-specific fluorescent dye such as QuantiFluor^®^ dsDNA Dye (Promega, Madison, WI, USA). The amount of PCR products depends on the quantity and integrity of the template, the same amount of template is used for each reaction, reaction with half the amount of template (5 ng) was used as a control for reaction. DNA methylating agent, MMS was used as a positive control for the experiment. The protocol was adopted from Santos et al. with modifications [59]. Logarithmic phase cell culture of W303-1A strain in SD medium was treated with compounds. After treatment, 5 mL of cell was collected (5 min, 4000× *g*), samples were incubated with 2 mg/mL Zymolyase 20T (Carl Roth, Karlsruhe, Germany) for 30 min at 30 °C to digest cell wall and DNA was extracted with Bacterial and Yeast Genomic DNA Purification Kit (EURx, Gdańsk, Poland) according to manufacturer’s instruction. DNA concentration of samples was adjusted to 10 ng/µL.

PCR reactions amplifying 6.9-kb mitochondria fragment in *COX1* gene were prepared as follows:

- 1 µL of 10 ng/µL template DNA
- 12.5 µL of LongAmp^®^ Taq 2X Master Mix (New England Biolabs, Ipswich, MA, USA)
- 10 µL of Nuclease-free water
- 1 µL of 10 µM Sense primer (Genomed, Warszawa, Poland), 5′-GTG CGT ATA TTT CGT TGA TGC GT-3′
- 1 µL of 10µM Antisense primer (Genomed, Warszawa, Poland), 5′-GTC ACC ACC TCC TGC TAC TTC AA-3′

PCR reaction was performed in 14 cycles. After initial denaturation for 30 s at 94 °C, each cycle comprised 30 s denaturation at 94 °C, 60 s annealing at 60 °C, 6 min extension at 65 °C, and with a final extension for 10 min at 65 °C at the end of the last cycle. QuantiFluor^®^ dsDNA System was used to measure the fluorescence intensity of samples. Fluorescence was measured with Thermo Scientific™ Varioskan™ LUX multimode microplate reader at 504nmEx/531nmEm. Additionally, samples were analyzed with agarose gel electrophoresis.

### 4.6. MTT Assay

Logarithmic phase cell culture of W303-1A and *rho*^0^ strains in SD medium were treated with 3-BP. After treatment OD600 of samples was adjusted to 0.5 and 1 mL of cells was centrifuged (1 min, 10,000× *g*) medium was removed and the cell pellet was resuspended in 1 mL of PBS containing 0.5 mg/mL MTT (Sigma-Aldrich^®^, St. Louis, MO, USA). Samples were incubated at 30 °C for 4 h, centrifuged (1 min, 10,000× *g*) supernatant was removed and 1 mL of DMSO was added to cells to solubilize formazan. Samples were incubated for 15 min with agitation in dark, centrifuged (1 min, 10,000× *g*) supernatant was removed transferred and absorbance was measured at 590 nm in NanoPhotometer^®^ NP80 (Implen, Munich, Germany).

## Figures and Tables

**Table 1:** Yeast strains used in this study.

Strain Name	Genotype	Source
W303-1A	*MAT*a *ade2-1 can1-100 ura3-1 his3-11,15 leu2-3, 112trp1-1 RAD5*	R. Rothstein
*rho* ^0^	W303-1A, *rho*^0^	This work
BY4741	*MAT a,* Δ*his3;* Δ*leu2;* Δ*met15;* Δ*ura3*	Euroscarf
Δ*mhr1*	BY4741, Δ*mhr1*	Euroscarf
Δ*ctt1*	BY4741, Δ*ctt1*	Euroscarf
Δ*pcp1*	BY4741, Δ*pcp1*	Euroscarf
Δ*gpx1*	BY4741, Δ*gpx1*	Euroscarf
Δ*ccp1*	BY4741, Δ*ccp1*	Euroscarf
Δ*hmi1*	BY4741, Δ*hmi1*	Euroscarf
Δ*sod2*	BY4741, Δ*sod2*	Euroscarf
Δ*mgm101*	BY4741, Δ*mgm101*	Euroscarf
Δ*por1*	BY4741, Δ*por1*	Euroscarf
Δ*rim1*	BY4741, Δ*rim1*	Euroscarf

## Data Availability

Not applicable.
